# Is It Fear? Similar Brain Responses to Fearful and Neutral Faces in Infants with a Heightened Likelihood for Autism Spectrum Disorder

**DOI:** 10.1007/s10803-020-04560-x

**Published:** 2020-06-27

**Authors:** Renata Di Lorenzo, Nicolette M. Munsters, Emma K. Ward, Maretha de Jonge, Chantal Kemner, Carlijn van den Boomen

**Affiliations:** 1 Experimental Psychology, Helmholtz Institute, Utrecht University, Langeveld Building, Heidelberglaan 1, 3584 CS Utrecht, The Netherlands; 2 Developmental Psychology, Utrecht University, Langeveld Building, Heidelberglaan 1, 3584 CS Utrecht, The Netherlands; 3 Department of Psychiatry, University Medical Center Utrecht Brain Center, Utrecht University, Utrecht, The Netherlands; 4 Karakter Child and Adolescent Psychiatry, Ede, The Netherlands; 5 Donders Institute for Brain, Cognition and Behaviour, Radboud University, Nijmegen, The Netherlands; 6 Clinical Neurodevelopmental Sciences, Leiden University, Leiden, The Netherlands

**Keywords:** Autism spectrum disorder, Infants, Spatial frequency, Emotion, Event-related potentials, Endophenotype

## Abstract

Individuals with autism spectrum disorder (ASD) show atypical processing of facial expressions. Research with autistic toddlers suggests that abnormalities in processing of spatial frequencies (SFs) contribute to such differences. The current event-related-potential (ERP) study investigated differences between 10-month-old infants with high- and low-likelihood for ASD in SF processing and in discrimination of fearful and neutral faces, filtered to contain specific SF. Results indicate no group differences in general processing of higher (HSF, detailed) and lower-SF (LSF, global) information. However, unlike low-likelihood infants, high-likelihood infants do not discriminate between facial expressions when either the LSF or HSF information is available. Combined with previous findings in toddlers, the current results indicate a developmental delay in efficient processing of facial expressions in ASD.

Difficulties in social communication are a prominent and defining characteristic of autism spectrum disorder (ASD; American Psychiatric Association 2013). It has been previously hypothesized that these behavioral difficulties in individuals with ASD relate to differences in the neural processing of social stimuli, such as facial expressions (Black et al. 2017; Elsabbagh and Johnson 2007). Atypical facial expression processing could arise from an even more basic visual process: the rather detailed-oriented perception that is often observed in individuals with ASD (e.g., Dakin and Frith 2005; Happé and Frith 2006: Vlamings et al. 2010). Previous research in 3- to 4-year-olds with ASD revealed both general enhanced brain activity in response to details as well as detail-driven facial expression processing (Vlamings et al. 2010). The current study aims to understand whether the visual abnormalities in (i) the processing of detailed information and (ii) the processing of facial expressions based on detailed information, are already present in infants with a heightened familial likelihood for an ASD diagnosis, and could thus be an endophenotype for ASD.

The detailed-oriented perception that is often observed in individuals with ASD might relate to differences in the processing of spatial frequencies (SFs; Boeschoten et al. 2007; Curby et al. 2003; Deruelle et al. 2004; Deruelle et al. 2008; Vlamings et al. 2010). Spatial frequency refers to the number of cycles of luminance variations (i.e. changes between dark and light) per degree of the visual angle an object subtends at the retina and is measured in cycles per degree (cpd) of visual angle. Higher spatial frequencies (HSFs, small-scale luminance variations, e.g. > 6 cpd) are suggested to play a central role in the encoding of detailed visual information such as sharp edges. Whereas lower spatial frequencies (LSFs, large-scale luminance variations, e.g. < 2 cpd) are suggested to be important for the processing of global configurations (Goffaux and Rossion 2006; Morrison and Schyns 2001). Figure 1 shows an example of faces that are filtered to contain only the HSF or LSF information. HSF and LSF are thought to be processed through different interconnected neural pathways (Carey and Diamond 1977; Johnson 2005; Johnson et al. 2015): it is hypothesized that HSF are carried by the slow-processing, cortical parvocellular pathway, while LSF are conveyed by the fast-processing, subcortical and cortical magnocellular pathways.

Atypical processing of SF in non-face stimuli in individuals with ASD has been shown from childhood onwards. Specifically, adolescents and adults with ASD show increased visual sensitivity to HSF information compared to neurotypical adolescents and adults (Kéïta et al. 2014). Atypical processing of HSF information is also observed in younger children with ASD, as indicated by differences in event-related potentials (ERPs; Boeschoten et al. 2007; Vlamings et al. 2010). For instance, the amplitude of the P100, an ERP component reflecting very early phases of visual processing, was found to be higher in response to HSF than LSF gratings in toddlers with ASD, while the opposite pattern was visible in toddlers in the control group (Vlamings et al. 2010). Whilst studies in children with ASD did not examine SF effects on later ERP components indexing later perceptual stages, findings in typically-developing children reveal that SF content also affects later components such as the N2 (van den Boomen et al. 2015). While these differences have been observed in preschool children, adolescents and adults, it is unclear whether SF processing is already atypical at an even younger age and at which stage of visual processing these differences can be detected. The current research is the first that explores SF processing in infants with a heightened familial likelihood for an ASD diagnosis.

The processing of facial expressions differs already between infants with higher familial likelihood for an ASD diagnosis (high-likelihood infants) and infants with lower familial likelihood for an ASD diagnosis (low-likelihood infants) (Key et al. 2015; Key and Stone 2012). Specifically, differences are revealed in the face-sensitive N290 and P400 ERP components, suggested to be the infant precursor of the adult N170 component (de Haan et al. 2003). Similar differences are also reported in child- and adulthood (for a review on ERP studies see Black et al. 2017). In particular, in child- and adulthood, group differences in the ERP responses to facial expressions are observed for the early visual P100 component as well as for the later visual face-sensitive N170 component. The atypical processing of facial expressions in ASD is suggested to be at least partly due to abnormalities in the processing of SF. For instance, children with ASD show increased use of HSF to process facial expressions compared to children without ASD (Deruelle et al. 2004, 2008; Vlamings et al. 2010). Notably, an ERP study indicated that in 3- to 4-year-olds with ASD the P100 amplitude differed between fearful and neutral expressions only when the face stimuli contained HSF (Vlamings et al. 2010). Children in the control group showed the opposite pattern: only in the LSF condition were there P100 amplitude differences between expressions. Note that no group differences were found for the later face-sensitive N170, which suggests that atypicalities in the use of SF to discriminate facial expressions appear at earlier phases of visual processing (Vlamings et al. 2010). However, it is unknown whether infants with a higher likelihood for an ASD diagnosis differ from infants with a lower likelihood in the use of spatial frequencies when processing facial expressions.

In sum, in the current study we aim to understand whether infants with high-likelihood (HL) and low-likelihood (LL) of developing ASD differ in (i) the general visual processing of HSF and LSF information; (ii) the processing of facial expressions when they selectively contain HSF or LSF information. To this end, we recorded cortical activity from 10-month-old HL and LL infants while they passively watched fearful and neutral faces, filtered to contain only HSF or LSF information. Based on previous results in toddlers with ASD and toddlers in the control group (van den Boomen et al. 2015; Vlamings et al. 2010), we hypothesize the HL group will show an atypical pattern of HSF versus LSF processing compared to the LL group regardless of the expression displayed, as indexed by the P100, N290, and P400 components.

With regard to the second aim of this study, we base our hypotheses of the LL group on recent ERP research with typically-developing 10-month-olds (van den Boomen et al. 2019). This study revealed that infants could discriminate between facial expressions when only HSF information was available, but not when only LSF information was available. However, the study had a slightly different design than the current one^1^: van den Boomen and colleagues presented happy expressions in addition to the current fearful and neutral ones, and did not investigate the P100 component. Amplitude differences at the N290 and P400 components were observed between happy and fearful or neutral expressions, but not between fearful and neutral ones. As such, we do not expect differences between fearful and neutral expressions at these later components in the current study, for any likelihood group. However, it is possible that differences between these expressions can be observed at the P100 component. Therefore, we hypothesize that for the LL group, the P100 will be sensitive to type of expression when faces contain only HSF but not when faces contain only LSF. For the HL group we hypothesize, based on Vlamings et al. (2010), an atypical pattern of activation at the P100 component.

## Methods

### Participants

Thirty-eight 10-month-olds participated in this study, of which 20 had at least 1 older sibling with a clinical diagnosis on the autism spectrum (HL group; diagnosis confirmed to researchers via a copy of the diagnostic report), and 18 had at least 1 older sibling without a clinical diagnosis on the autism spectrum, and no family history of clinical diagnosis on the autism spectrum (LL group; based on parent report; see Table 1). The sample size is comparable to previous ERP studies on facial expression processing in infancy (e.g., Hoehl and Striano 2010; Leppänen et al. 2007) as well as on SF and expression processing in childhood (e.g., Vlamings et al. 2010). All included infants were born full term (> 36 weeks). During the visit, the Mullen Scales of Early Learning (MSEL; Mullen 1995) was also administered. HL and LL infants did not differ in chronological age or developmental level as measured by the MSEL Composite score (see Table 1). An additional fifty-two infants were excluded due to an insufficient number of valid trials due to experimental error (9 infants), no EEG data being acquired (6 infants), insufficient looking to screen (2 infants), or high level of artifacts in the data (35 infants). The HL infants were recruited via collaborations with practitioners and via Dutch patients and parent associations. The LL infants were recruited from the participant databases of Utrecht University and the Radboud University Nijmegen. The current study was embedded in a longitudinal multi-centre study looking at the early development of autism (EU-AIMS project; see Loth et al. 2017). Infants were tested at one of two sites (A or B); the testing procedure was identical unless otherwise noted. Of the 38 infants, 30 infants were tested at site A (14 HL and 16 LL) and 8 infants at site B (6 HL and 2 LL). The project at both sites was approved by the Medical Ethical Committee of the Arnhem-Nijmegen Region (protocol NL42726.091.13). The study was performed in accordance with the Helsinki Declaration. Parents signed informed consent prior to participation and received monetary compensation for their time, travel costs when applicable, and a small present for the child. At the time of writing, we do not have complete information on the diagnostic outcome of the sample.

**Table 1:** Characteristics of the participants included in the analysis

	HL	LL	*t*(36)	*p*
N	20 (8♀)	18 (9♀)		
Age in days (SD)	308 (22.6)	311 (16.2)	0.47	.64
Range age in days	257–344	274–345		
Viewed trials (SD)	106 (16.8)	116 (20.4)	1.64	.11
MSEL-ELC (SD)	92.1 (12.3)	94.9 (10.2)	0.74	.46

We verified that the groups were similar in age, number of viewed trials, and developmental stage as measured by the MSEL Composite Score

### Stimuli

**Fig. 1:**
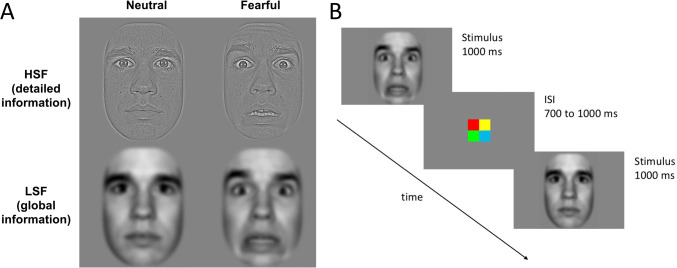
**a** Examples of the neutral (first column) and fearful (second column) face stimuli, filtered to contain higher spatial frequency (HSF, detailed information, first row) or lower spatial frequency (LSF, global information, second row). Similar example of stimuli were first shown in van den Boomen et al. (2019). **b** Example of the experimental design showing the timing of stimuli and ISI presentation

Face stimuli were photographs of 10 models each expressing a neutral and a fearful expression, which were taken from the MacBrain Face Stimulus Set.^2^ The pictures included 5 males and 5 females, of which 6 European-American, 3 African-American and 1 Asian-American model. Using Photoshop, face pictures were trimmed to remove external features (neck, ears and hairline), all stimuli were cropped, turned into grey scale and matched for size (19.4 × 14.0° of visual angle at a viewing distance of 65 cm). All faces were filtered with a low- (LSF; < 2 cycles per degree) or high-pass (HSF; > 6 cycles per degree) spatial frequency filter. The LSF and HSF stimuli differed in terms of root mean square (RMS) contrast (LSF: 25 cd/m^2^; HSF: 8 cd/m^2^). Stimuli were presented on a grey background (RGB: 131 × 131 × 131). Taken together, this created a 2 (expression: neutral, fearful) × 2 (SF: LSF, HSF) condition design (Fig. 1).

### Procedure

Infants were visited at home by the research team (site A) or invited to the lab (site B). When testing took place at home (site A), a tent was placed over the dining table to approximate equal lighting conditions for each measurement. The tent comprised fabric in front, to the left and right sides, and above the child to block surrounding visual distractions. The back of the tent was left open, so parents or experimenters could move closer to the child if they were too fussy or showed discomfort. At site B, the infant and one parent were seated in a Faraday-shielded testing booth. At both sites, the infant sat on a high-chair or on the parent’s lap at approximately 65 cm distance from a 23-in. computer monitor (refresh rate 60 Hz, 1920 × 1080 resolution). Parents were instructed to prevent interaction with their child as much as possible during the experiment, but to stop the child from pulling the cables. In addition, the parents were instructed that if interaction was required the parent could hold the child or the child’s hand, but not stroke or bounce the child, and to respond with sentences such as ‘I see the screen’ but not name items on or point to the screen. The face stimuli were presented in two blocks of 20 stimuli per condition in random order for 1000 ms, with a jittered interstimulus interval between 700 and 1000 ms. The interstimulus interval contained a square of 202 by 202 pixels, divided into four coloured square of equal sizes. The colours were red (RGB: 255 × 0 × 0; left top), yellow (RGB: 255 × 255 × 0; right top), green (RGB: 0 × 255 × 0; left bottom), and blue (RGB: 0 × 185 × 233; right bottom). A video camera placed near the screen recorded the child’s behaviour during the testing session. When the infant disengaged from the screen, the experimenter paused the task and reoriented the infant’s attention by playing additional sounds or a moving stimulus on the screen. Unattended trials were discarded from analyses. The average number of attended trials was 106 for the HL group and 116 for the LL group (see Table 1). The experiment ended when all 160 trials were presented or until the child was too distracted or fussy to attend. The task lasted approximately 5 min (excluding breaks and attention reorientation).

### Data Analyses

#### ERP Recording

Two different EEG systems were used to record brain activity at the two testing-sites. At site A, EEG data was acquired using the 32-channel ActiveTwo BioSemi system (Amsterdam, Netherlands); electrodes were positioned at standard EEG recording locations according to the international 10–20 system. During the experiment, continuous EEG was acquired at a 2048 Hz sample rate using Actiview (version 7.05). Two electrodes, CMS (Common Mode Sense) and DRL (Driven Right Leg) provided an active ground. At site B, EEG was recorded from 32 AgCl active electrodes in the 10–20 configuration, in a child-sized EEG cap (ActiCap, Brain Products, Munich, Germany) using BrainVision Recorder, via a BrainAmp BrainVision Products amplifier. EEG was recorded continuously, with an online reference at FCz, at a sampling frequency of 500 Hz and with a band-pass filter (0.1–125 Hz).

#### Preprocessing

EEG data were pre-processed using Brain Vision Analyser software (version 2.1; Brainproducts, GmbH). Data were resampled offline to 512 Hz, and filtered with a high-pass filter of 0.1 Hz (24 dB/oct), a low-pass filter of 30 Hz (24 dB/oct) and a notch filter of 50 Hz. Continuous EEG data were first divided into epochs of 100 ms pre-stimulus (baseline) until 1000 ms post-stimulus and then demeaned with baseline defined as 100 ms pre-stimulus until stimulus onset. Videos of the experimental session were manually coded for looking behaviour, and trials were removed in all electrodes if the child blinked or looked away between 0 and 500 ms after stimulus onset. Artifacts were defined as amplitudes + / − 200 µV; as a difference of less than 3 µV within 200 ms; or as a voltage change of more than 50 µV from the previous sampling point. An electrode was rejected if there were less than five artifact-free trials. We removed trials when more than 5 electrodes (16% of the total number of electrodes) contained artifacts, based on previous research on face processing in infants (see van den Boomen et al. 2019). Finally, the activity of each electrode was re-referenced to the average of all included electrodes.

Participants were included in the statistical analyses if the final average per experimental condition contained at least 10 trials per condition for critical electrodes (i.e., P7, P3, O1, Oz, O2, P4, P8). The average number of included trials was 24.7 per condition (*SD* = 0.51, range = 24.2–25.3).

#### Component Analyses

The components of interest were the P100, N290 and P400. Mean amplitude within a time window of 120–240 ms (P100), 240–340 ms (N290) and 340–600 ms (P400) was used to compute statistical analyses on these components. For each component, electrodes of interest were P7, P3, O1, Oz, O2, P4, P8; our choice of electrodes was based on previous similar research (e.g., Guy et al. 2018; Munsters et al. 2019; Vlamings et al. 2010) and the presence of peaks in these electrodes was confirmed by visual inspection. Mean amplitude was averaged over all electrodes of interest for each component to limit the number of statistical comparisons.

#### Statistical Analyses

Statistical tests were performed using IBM SPSS Statistics 25.0 (IBM Corporation, Armonk, NY, USA). For the amplitude of each component, we investigated whether HL and LL infants differed in (i) general SF processing, and (ii) facial expression processing based on specific SF. Thus, we planned to compute a three-way repeated measures ANOVA with SF (HSF vs. LSF) and Expression (neutral vs. fearful) as within-subject factors, and Group (HL vs. LL) as a between-subjects factor. Whereas an interaction between Group and SF indexes group difference in the general processing of SF (Hypothesis I), a three-way interaction indicates that differential facial expression processing is based on different SF information for HL vs. LL infants (Hypothesis II).

However, for the P100, one variable (i.e. the P100 amplitude evoked by LSF fearful faces in the HL group) was not normally distributed. Therefore, non-parametric tests would be the best choice for this data. Therefore, we performed non-parametric tests on planned comparisons. The planned comparisons included the following: (1) to investigate facial expression discrimination based on specific SF, we tested the effects of Expression for each of the SF conditions per group using Wilcoxon signed rank tests; (2) testing for SF effects within each Group and Expression (Wilcoxon signed rank tests); and (3) testing group effects within each SF and Expression (Mann–Whitney U tests). It is noteworthy that the last two tests are rather exploratory as they do not directly answer our specific research questions, but are provided as additional information; hence, we did not correct the results of the last two tests for multiple comparisons. Furthermore, the three analyses were conducted non-parametrically for both groups. Note that, even though only in the HL group the data was not normally distributed, we chose non-parametric testing for analyses on both groups instead of only the HL group to ensure equal statistical power of analyses in each group.

To investigate general SF processing at the P100 (Hypothesis I), we tested the effects of SF on the cortical activity collapsed over facial expressions (i.e. mean activity of both facial expressions). Because the collapsed activity over facial expressions was normally distributed in both groups, this analysis could be conducted parametrically. As such we conducted a repeated measures ANOVA with SF as within and Group as between subject variable, that was possibly followed-up by t-tests.

For the N290 and P400 components, all data was normally distributed and therefore the above-described three-way ANOVA was performed for each of these components. For all reported analyses, the alpha value was set at 0.05. When appropriate, Bonferroni correction for multiple testing was applied.

## Results

### P100 Amplitude

**Fig. 2:**
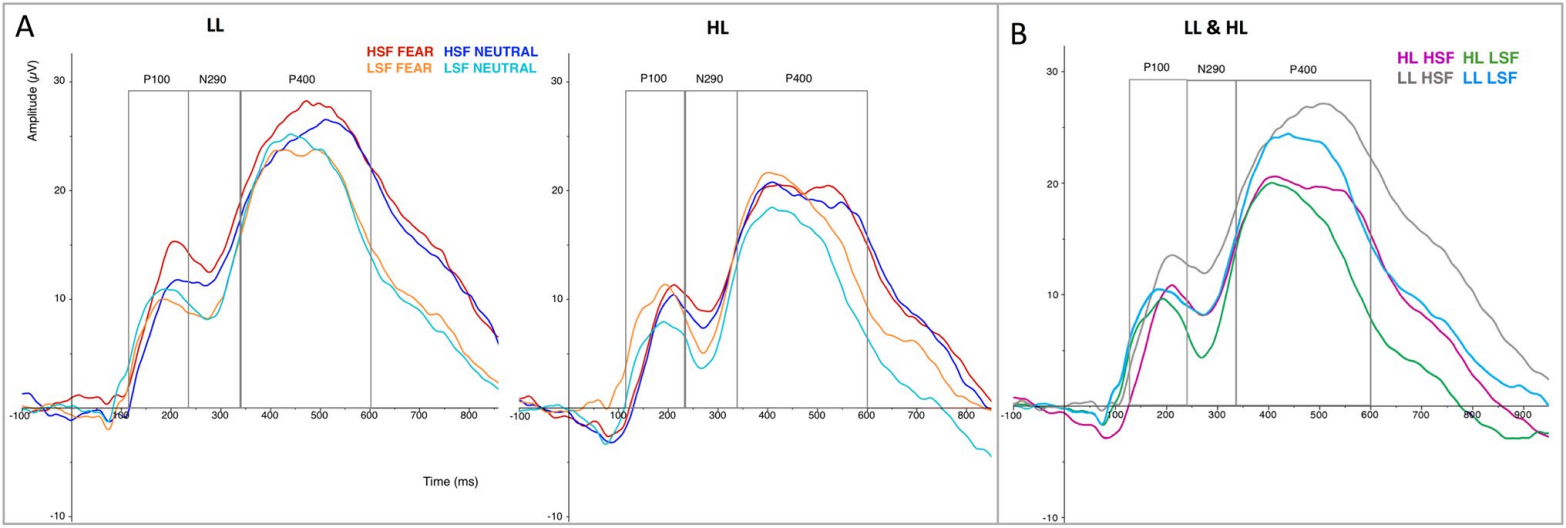
Grand averages of the HL and LL group, at the pooled electrodes. **a** Brain responses to all conditions for LL (left panel) and HL (right panel) infants. **b** Responses of both infant groups to HSF and LSF (i.e. averaged across expressions). Rectangles indicate the time windows chosen for the P100, N290 and P400 components

**Fig. 3:**
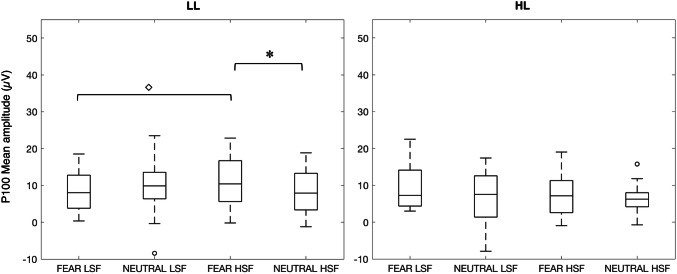
Box plots of the P100 mean amplitudes obtained for each condition in the LL (left panel) and HL group (right panel). The horizontal line in the box plots indicates the median, while the two whiskers denote the first and third quartile. Outliers are represented with circles. The asterisks above the lines linking the different conditions indicate significant statistical differences, Bonferroni corrected; while the diamond indicates significant results that are non-corrected

Figure 2 depicts the grand averages, and Fig. 3 the boxplots of the mean amplitudes for the P100 of the HL and LL group.

The two-way ANOVA yielded no significant interaction between SF and Group [*F*(1,36) = 1.40, *p* = 0.25, η_p_^2^ = 0.037], indicating no substantial group differences in general SF processing at the P100 amplitude. There was no main effect of SF [*F*(1,36) = 0.71, *p* = 0.79, η_p_^2^ = 0.002], nor of group [*F*(1,36) = 1.42, *p* = 0.24, η_p_^2^ = 0.038].

The Wilcoxon signed rank tests performed to understand whether HL and LL infants showed differential processing between Expressions based on specific SF revealed that only for the LL group HSF fearful faces elicited a significantly larger P100 amplitude (*Mdn* = 10.4) than HSF neutral faces (*Mdn* = 7.90), *Z* = 2.37, *p* = 0.018, *r* = 0.56. No difference between fearful and neutral faces was found for the LL group in the LSF condition (*Mdn* Fearful = 7.99; *Mdn* Neutral = 9.85; *Z* = 0.022, *p* = 0.98, *r* = 0.005), nor for the HL group in either SF condition (HSF: *Mdn* Fearful = 7.14; *Mdn* Neutral = 6.24; *Z* = 0.71, *p* = 0.48, *r* = 0.16; LSF: *Mdn* Fearful = 7.27; *Mdn* Neutral = 7.53; *Z* = 2.02, *p* = 0.044, *r* = 0.45; against alpha of 0.025 corrected for two tests per group).

To further explore any group differences in processing the stimuli, we tested (i) possible SF effects within each Group and Expression (Wilcoxon signed rank tests); and (ii) possible Group effects within each SF and Expression (Mann–Whitney U tests). The Wilcoxon signed rank tests comparing responses to HSF vs. LSF information in Neutral faces revealed no effect of SF for either the HL (*Z* = 0.075; *p* = 0.94) nor the LL group (*Z* = 1.68; *p* = 0.094). For Fearful faces, there was no effect of SF in the HL group (*Z* = 1.34; *p* = 0.18), while there was a higher amplitude evoked by HSF (*Mdn* = 10.4) than LSF (*Mdn* = 7.99) in the LL group (*Z* = 2.29, *p* = 0.022, *r* = 0.54). The Mann–Whitney U tests revealed no group difference for any SF and Expression (all *p*s > 0.09). Note that due to the exploratory nature of these tests, we report results uncorrected for multiple testing.

Overall these analyses indicate no group differences in general SF processing, but suggest that when Expression is taken into account more subtle differences can be observed. That is, a larger P100 amplitude might be evoked by HSF than LSF fearful faces in the LL but not in the HL group.

### N290 Amplitude

**Fig. 4:**
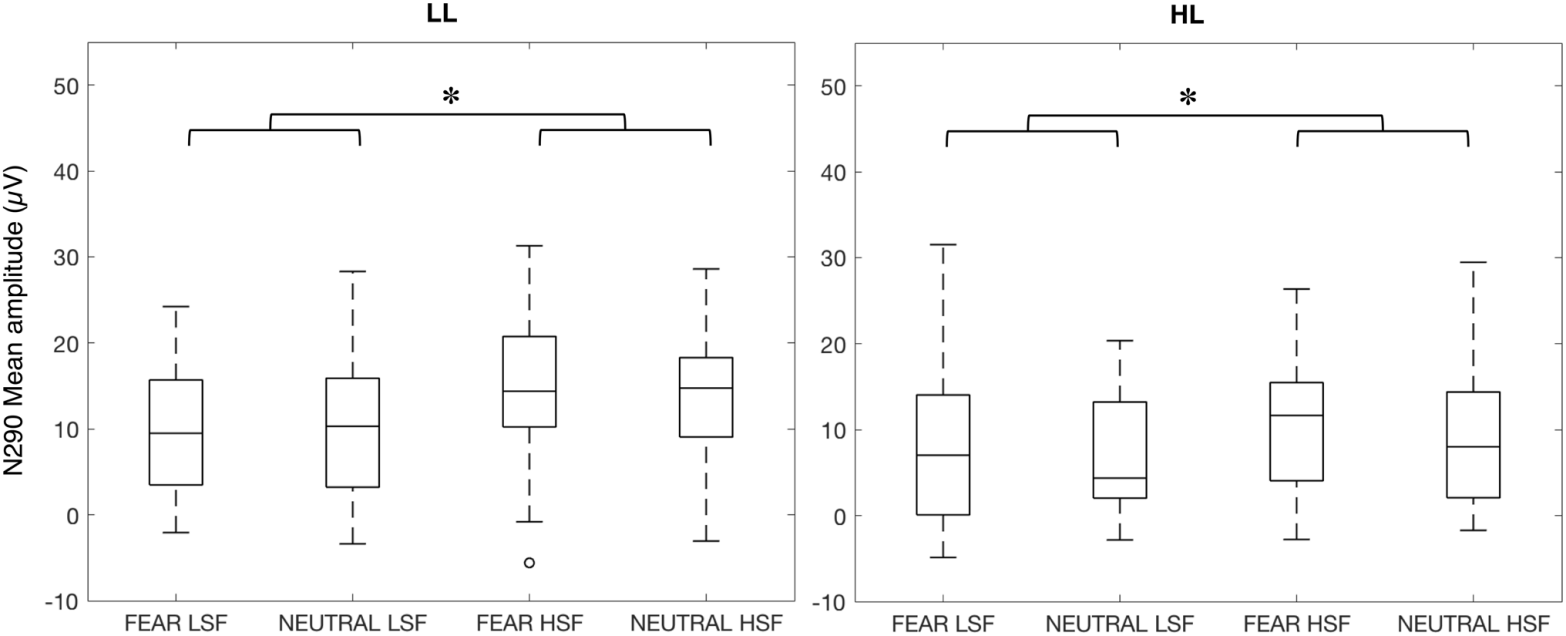
Box plots of the N290 mean amplitudes obtained for each condition in the LL (left panel) and HL group (right panel). The horizontal line in the box plots indicates the median, while the two whiskers denote the first and third quartile. Outliers are represented with circles. The asterisks above the lines linking the different conditions indicate significant statistical differences, Bonferroni corrected

Figure 2 depicts the grand averages, and Fig. 4 the boxplots of the mean amplitudes for the N290 of the HL and LL group. The three-way ANOVA revealed a main effect of SF [*F*(1,36) = 10.5, *p* = 0.003, η_p_^2^ = 0.23]: LSF evoked a more negative response (*M* = 8.76; *SD* = 8.54) than HSF stimuli (*M* = 11.8; *SD* = 8.40). The analyses did not show any other main or interaction effect: there was no significant three-way interaction [*F*(1,36) = 1.09, *p* = 0.30], nor any further effects including SF, Expression, or Group (all *ps* > 0.14). These analyses indicate that there were no substantial group differences in general SF processing and in facial expression processing based on specific SF at the N290 amplitude.

### P400

**Fig. 5:**
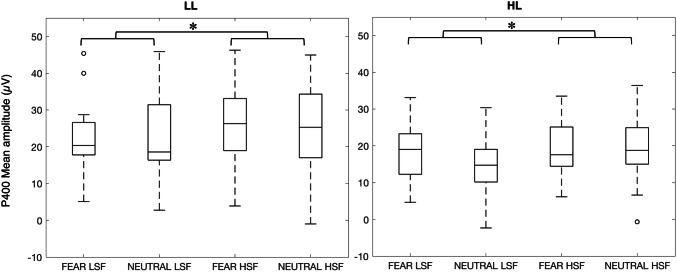
Box plots of the P400 mean amplitudes obtained for each condition in the LL (left panel) and HL group (right panel). The horizontal line in the box plots indicates the median, while the two whiskers denote the first and third quartile. Outliers are represented with circles. The asterisks above the lines linking the different conditions indicate significant statistical differences, Bonferroni corrected

Figure 2 depicts the grand averages, and Fig. 5 the boxplots of the mean amplitudes for the P400 of the HL and LL group. Similarly to our findings for the N290, the three-way ANOVA revealed a main effect of SF [*F*(1,36) = 9.55, *p* = 0.004, η_p_^2^ = 0.21]: LSF evoked a lower amplitude (*M* = 19.0; *SD* = 8.91) than HSF stimuli (*M* = 21.9; *SD* = 9.75). Again, there was no interaction of Group and SF [*F*(1,36) = 0.06, *p* = 0.81], indicating that there were no substantial group differences in general SF processing at the P400 amplitude.

However, for this ERP component the ANOVA revealed a three-way interaction [*F*(1,36) = 4.23, *p* = 0.047, η_p_^2^ = 0.10]. As a follow-up, we conducted two-way ANOVAs which revealed that two-way interactions were not significant: (1) per group there was no interaction between SF and Expression [HL: *F*(1,19) = 2.44, *p* = 0.13; LL: *F*(1,17) = 1.91, *p* = 0.18]; (2) per SF there was no interaction between Expression and Group [LSF: *F*(1,36) = 2.59, *p* = 0.12; HSF: *F*(1,36) = 0.52, *p* = 0.47]; (3) per Expression there was no interaction between SF and Group [Fearful: *F*(1,36) = 2.45, *p* = 0.13; Neutral: *F*(1,36) = 0.57, *p* = 0.45]. However, these two-way ANOVAs did show relevant main effects. In particular, the above-reported main effect of SF also emerged from the two-way ANOVAs split by Expression [analysis 3; Fear: *F*(1,36) = 8.96, *p* = 0.005; Neutral: *F*(1,36) = 5.93, *p* = 0.020]: in both expressions, HSF evoked larger amplitudes than LSF. Furthermore, the two-way ANOVA split by Group (analysis 1) showed this SF main effect for the LL group [*F*(1,17) = 5.84, *p* = 0.027, *M* LSF = 21.6, *M* HSF = 24.7], but only at trend level for the HL group [*F*(1,19) = 3.94, *p* = 0.062, *M* LSF = 16.4, *M* HSF = 19.1]. All two-way ANOVAs did not show any main effects of Expression or Group (all *ps* > 0.05). For these exploratory analyses we report uncorrected p-values tested against an alpha of 0.05.

Even though there were no two-way interactions, we here report the results of the pairwise comparisons to guide future research. These analyses are not validated by the above-described results and should therefore be interpreted with much caution. For the LL group, there was higher amplitude evoked by HSF than LSF fearful but not neutral faces [Fearful: *t*(17) =  − 3.6; *p* = 0.002; Neutral: *t*(17) =  − 1.2; *p* = 0.240]. Furthermore, there was no difference evoked by fearful versus neutral faces in either the LSF or HSF condition [LSF: *t*(17) = 1.4; *p* = 0.177; HSF: *t*(17) =  − 0.2; *p* = 0.810]. For the HL group, there was a higher amplitude evoked by HSF than LSF neutral but not fearful faces [Fearful: *t*(19) =  − 0.9; *p* = 0.355; Neutral: *t*(19) =  − 2.2; *p* = 0.038]. Furthermore, there was a higher amplitude evoked by fearful than neutral faces for the LSF but not HSF condition [LSF: *t*(19) = 2.1; *p* = 0.047; HSF: *t*(19) = 0.2; *p* = 0.836]. Finally, comparing groups revealed a larger amplitude evoked by neutral LSF faces in the LL than HL group [*t*(36) = 2.2; *p* = 0.036], but no other group differences [neutral HSF: *t*(36) = 1.5; *p* = 0.141; fearful LSF: *t*(36) = 1.2; *p* = 0.249; fearful HSF: *t*(36) = 1.9; *p* = 0.061]. Again, for these exploratory analyses we report uncorrected p-values tested against an alpha of 0.05.

Overall, the three-way ANOVA with follow-up tests reveal a three-way interaction between Group, SF, and Expression, suggesting that at some level facial expression processing based on specific SF for LL and HL infants differ. However, the follow-up analyses do not provide a clear explanation for this interaction but might suggest subtle differences in processing of the facial stimuli.

## Discussion

Previously, it has been suggested that the rather detailed-oriented perception that is often observed in individuals with ASD might contribute to difficulties in facial expression processing (e.g., Dakin and Frith 2005; Happé and Frith 2006; Vlamings et al. 2010). Yet, little is known on visual perceptual differences in infants with a heightened familial likelihood of developing ASD. In the present study, we examined whether 10-month-old infants with a higher familial likelihood for an ASD diagnosis (HL) already differ from infants with lower familial likelihood for an ASD diagnosis (LL) in (i) the general visual processing of HSF and LSF information, and (ii) in processing facial expressions when they selectively contain HSF or LSF information. We measured visual ERPs in response to neutral and fearful facial expressions, filtered to contain only HSF or only LSF information. We analysed group differences in SF processing for the mean amplitude of each component of interest (i.e. P100, N290, P400) in response to all faces regardless of the emotional content. The results of these analyses revealed no substantial difference between HL and LL infants in SF processing. That is, both groups showed similar P100 amplitudes for HSF and LSF stimuli, and lower N290 and P400 amplitudes for LSF than HSF stimuli. Yet, exploratory analyses on the P100 indicate more subtle group differences: only in the LL but not the HL group did HSF elicit larger amplitudes than LSF (specifically in the fearful condition). Crucially, we examined differences between HL and LL infants in the use of SF when processing facial expressions, as indexed by the amplitude of the early P100. Consistent with our hypothesis, our results suggest that the LL infants discriminated between facial expressions only when HSF were displayed, whereas the HL group did not differentiate between expressions in either SF condition. When we tested expression discrimination for the two later components, i.e. the N290 and the P400, there was an interaction between group, facial expression, and spatial frequency at the P400 that seemed to be due to subtle processing differences. However, we did not find a clear indication of differential processing of facial expressions for any group and any SF condition, which supports previous findings with typically developing infants (van den Boomen et al. 2019) and extends them to the HL population.

Our analyses on SF discrimination in infancy, combined with results from previous work in older children, suggest differences in the developmental trajectories between LL children and HL children who receive an ASD diagnosis later on. Specifically, our study indicates that LL infants have more positive amplitudes evoked by HSF than LSF information (i.e. exploratory tests revealed in the P100 fearful condition, and planned analyses in the N290 and P400 averaged across expressions). This pattern is similar to the cortical patterns of typically-developing infants at 1 year of age (for a review see van den Boomen et al. 2012), but opposite to 3- to 15-year-olds’ patterns (van den Boomen et al. 2015; Vlamings et al. 2010). As such, children in the control (LL) group exhibit a reversal of the ERP patterns evoked by HSF and LSF between infancy and toddlerhood. However, there is no such reversal in the ERP pattern when comparing HL infants to toddlers with ASD (Vlamings et al. 2010): HL infants show either no robust difference (P100 component) or more positive amplitudes for HSF than LSF stimuli (N290 and, although less strongly, P400 components), and the latter is also observed in toddlers with ASD (Vlamings et al. 2010). Possibly, the reversal of neural responses to SF in children in the control (LL) group relates to changes in visual sensitivity to SF across development: behavioural studies revealed that sensitivity to HSF develops more rapidly during the first years of life compared to LSF (for a review see van den Boomen et al. 2012). The changes in sensitivity and the reversal in neural responses might reflect a development of the neural pathways involved in HSF and LSF processing, which are suggested to be the parvocellular and magnocellular pathways, respectively (Carey and Diamond 1977; Johnson 2005; Johnson et al. 2015). The deviating developmental trajectory of ERP patterns when comparing HL infants from the current study to previously studied ASD toddlers (Vlamings et al. 2010) might be seen as support for theories suggesting a disruption in these neural pathways (e.g., Johnson 2005; Schultz 2005). Yet, longitudinal studies following HL infants throughout the first years of life are required to confirm the absence of a reversal in ASD and to fully understand when SF processing diverges from that of typically-developing children.

Furthermore, our study suggests that HL and LL infants differ in the use of SF when processing facial expressions. Specifically, our results at the P100 component indicate that the LL infants discriminated between facial expressions only when HSF were displayed, whereas the HL group did not differentiate between expressions in either SF condition. Comparing these results to the literature suggests that children with ASD have a delayed development of the use of HSF information. As hypothesized, in the LL infants, facial expression discrimination seemed to be driven by HSF information at very early phases of visual processing (i.e., P100). This finding is consistent with previous work on later processing stages of facial expression discrimination in typically-developing infants (Jessen and Grossmann 2017; van den Boomen et al. 2019). Note that previous infant studies did not test SF effects on the P100 component. Conversely, for the HL group we found an indication of similar cortical responses to neutral and fearful faces in both SF conditions. These results provide the first evidence that HL and LL infants might differ in the processing of facial expressions when only LSF or only HSF information is available. Notably, a previous study showed that the ERP pattern of toddlers with ASD differed between facial expressions when HSF faces were displayed (P100; Vlamings et al. 2010), a pattern similar to that of the LL group of the current study. In that study, the ERP responses of toddlers in the control group differed between expressions when LSF faces were displayed (Vlamings et al. 2010). Thus, while for children in the control group facial expression discrimination seems to be based on HSF information at 10 months and on LSF information at 3 years, expression discrimination seems not to be present in HL infants at 10 months and is based on HSF in 3-year-olds with ASD. These developmental patterns might signal delayed development of facial expression discrimination in ASD. A delayed development of specific processes has been previously reported in children with ASD. For instance, ERPs evoked by faces in 18- to 30-month-olds with ASD resembled ERPs observed in typically-developing 12- to 17-month-olds, but differed from age-matched typically-developing children (Webb et al. 2011). It is unknown whether these delays in development resolve, such that processing of faces and facial expressions differs less from typically-developing individuals later on in development. Prior research with HL infants suggests that delayed development is resolved in some domains in ASD (e.g., grasping and reaching, Libertus et al. 2014; face processing, Jones et al. 2016) or that the presence of differences fluctuates across development (e.g., work on motor delays by Estes et al. 2015). Thus, it is difficult to predict whether a delayed development in facial expression discrimination will turn into a typical use of LSF information to process facial expressions later on individuals with ASD.

A delay in the development of processing facial expressions filtered to contain only HSF or LSF information early in life might affect the efficiency of expression discrimination. This could be due to the neural pathways via which HSF and LSF are processed in the brain (Johnson 2005, 2015). Recall the slow-processing cortical parvocellular route is thought to carry mainly HSF information, whereas the fast-processing subcortical and cortical magnocellular routes is thought to operate on LSF information. Thus, using HSF information might lead to slower facial expression discrimination than using LSF information. As such, using HSF information to discriminate between facial expressions, as observed in toddlers with ASD (Vlamings et al. 2010), might result is slower facial expression discrimination. This could also impact other aspects of social development, e.g. social interaction. Notably, a widely accepted account of human brain development referred to as neuroconstructivism posits that seemingly small differences early in life could accumulate into cascading effects in several domains over development (D’Souza and Karmiloff-Smith 2016). For example, the later onset of the ability to efficiently process facial expressions (i.e. via LSF information) might lead to difficulties in interpreting the social environment and consequently to differences in social behavior. In turn, this could affect the behavior of parents and peers, providing the child with different social input. Eventually, this cascade of alterations could contribute to difficulties in effective social communication.

While interpreting the current results, one should take into account that no information on diagnostic outcome is currently available for our sample. Consequently, one should not interpret our results as early signs of ASD since only a part of the HL group (approximately 20%, cf. Ozonoff et al. 2011) will develop ASD. Rather, our results indicate that differences in processing facial expressions when only LSF or HSF information is available could be an endophenotype of ASD, that is, an example of a subclinical autism trait observed at a neurocognitive level. Future research should test whether the differences reported here are evident in those HL infants who do later receive a diagnosis of ASD. In addition, an absence of effects, such as in the post-hoc analyses of the P400 component, might be due to the sample size. The current sample size was comparable to other infant EEG studies, but nevertheless could have been too small for the possibly more subtle effects to reach significance. As such, it is important that future studies with larger sample sizes replicate the current findings and again investigate possible effects at the N290 and P400. Furthermore, it is important to mention that here we tested only one specific expression contrast, i.e. fearful vs. neutral faces, hence our results cannot be generalized to all expression comparisons; further studies including different facial expressions are needed. In addition, future studies should follow HL infants across the first years of life to understand how sensitivity to SF in facial expressions as well as in non-social stimuli develops in ASD, and whether atypical SF processing affects later social development. Furthermore, only limited conclusions can be drawn regarding face processing of real-life faces. The current stimuli were filtered to contain specific spatial frequency information and therefore different from real-life, unfiltered, faces. Moreover, manipulating spatial frequencies resulted in changes in luminance contrast of the stimuli as well. As discussed by van den Boomen et al. (2019), this contrast change does not account for effects of spatial frequencies on processing of emotions in adults (Vlamings et al. 2009). It should be noted however that both contrast sensitivity and SF processing develop throughout childhood. Therefore, possible effects of contrast differences between spatial frequency conditions in the current study cannot be excluded.

Overall, the current study showed that HL and LL infants do not differ in general SF processing, but they seem to differ in the processing of facial expressions when only LSF or HSF information is available. That is, unlike LL infants, HL infants seemed unable to discriminate between facial expressions when only LSF or HSF information is available. Adding the current results to previous findings in toddlers with ASD (Vlamings et al. 2010) suggests a developmental delay in efficient processing of facial expressions in ASD.

## Footnotes

1 Note that the current study is part of a longitudinal project which started before the study of (van den Boomen et al. 2019) was published; therefore, our choice of facial expression conditions, that is, fearful and neutral expressions, was based on the work done with ASD children (Vlamings et al. 2010).

2 Development of the MacBrain Face Stimulus Set was overseen by Nim Tottenham and supported by the John D. and Catherine T. MacArthur Foundation Research Network on Early Experience and Brain Development. Please contact Nim Tottenham at tott0006@ tc.umn.edu for information concerning the stimulus set.
